# Comments to the “Letter to the Editor” for the manuscript titled “Increased expression of inflammasome signaling genes and proteins in selective brain regions in the intermediate stage of Alzheimer's disease”

**DOI:** 10.1111/bpa.70113

**Published:** 2026-06-07

**Authors:** Juan Pablo de Rivero Vaccari, David A. Davis, Andrew P. Sawaya, Susanna P. Garamszegi, Xiaoyan Sun, Ayled Barreda, Helen M. Bramlett, Sakir Humayun Gultekin, W. Dalton Dietrich, Robert W. Keane, Regina T. Vontell

**Affiliations:** ^1^ Department of Neurological Surgery and The Miami Project to Cure Paralysis University of Miami Miller School of Medicine Miami Florida USA; ^2^ Department of Cellular Physiology and Molecular Biophysics University of Miami Miller School of Medicine Miami Florida USA; ^3^ Department of Neurology University of Miami Brain Endowment Bank, University of Miami Miller School of Medicine Miami Florida USA; ^4^ Department of Neurology University of Miami Miller School of Medicine Miami Florida USA; ^5^ Dr. Phillip Frost Department of Dermatology and Cutaneous Surgery University of Miami Miller School of Medicine Miami Florida USA; ^6^ Department of Research Service Bruce W. Carter Department of Veterans Affairs Medical Center Miami Florida USA; ^7^ Department of Pathology University of Miami Miller School of Medicine Miami Florida USA; ^8^ Center for Cognitive Neuroscience and Aging University of Miami Miller School of Medicine Miami Florida USA

**Keywords:** Alzheimer's disease (AD), apoptosis‐associated speck‐like protein containing a caspase recruitment domain (ASC), endosomal sorting complexes required for transport (ESCRT), gasdermin‐D (GSDMD), ninjurin1 (NINJ1), NOD‐like receptors (NLRs), pyroptosis

## Abstract

Beta amyloid diffuse plaques, neurofibrillary tangles and neuritic plaques, are increased in densities at the intermediate stage of Alzheimer's neuropathological change. These pathological changes releasing Pathogen‐Associated Molecular Patterns (PAMPs) and Damage‐Associated Molecular Patterns (DAMPs). These molecules are sensed by pattern recognition receptors (PRRs) and trigger intracellular responses. One response is the activation of the inflammasome sensors NLRP1, NLRP3, and AIM2 to oligomerize with ASC speck to form the inflammasome complex and initiate the downstream signaling of GSDMD mediated pyroptosis. Another response is the increase in genes to manufacture proinflammatory cytokines, the inflammasome formation activates the cleavage of the proinflammatory cytokines to the activated forms, which are secreted into the extracellular environment and recruit a widespread inflammation

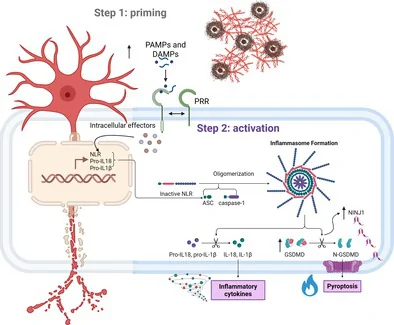

We thank the authors, Boa et al., for their insightful commentary, in the Letter to the Editor titled, “Hippocampal inflammasome enrichment in intermediate Alzheimer's disease: Strong spatial findings, but clearer stage and mechanistic definitions are needed,” about the manuscript titled, “Increased expression of inflammasome signaling genes and proteins in selective brain regions in the intermediate stage of Alzheimer's disease.” We would like to provide the following insight based on their letter.

In the study of de Rivero Vaccari et al. [1] we assessed how the inflammasome and inflammatory signals prorogate and alter different brain regions using samples from postmortem brains with low Alzheimer's disease (AD) neuropathological change and cases with intermediate AD neuropathological change to characterize age‐related inflammatory responses. We also compared cell type and brain region distribution of inflammasome and AD canonical targets in individuals diagnosed with mild to moderate cognitive changes.

Intermediate AD neuropathological change encompasses Braak stages III–V [2]. To control for the vast clinical and pathological changes identified with intermediate AD staging, we used mainly cases with Braak III and IV (e.g., one case was classified as Braak V). The staging differences between Braak III and Braak IV, showed more neurofibrillary tangles (NFTs) seen in frontal, temporal and occipito‐temporal cortical regions and a severely affected amygdala and hippocampus with a heavier burden of amyloid plaques (Aβ), NFTs, neuritic plaques (NPs) and reactive microglia. In the hippocampal CA2 region, the highly populated pyramidal cells show NFTs and NPs formation during the intermediate progression of AD pathology. This strategic selection ensures that our findings capture the period when neurofibrillary tangles (NFTs) and neuritic plaques (NPs) are actively expanding into the temporal and frontal regions, providing a clearer picture of regional inflammasome recruitment—We concur with that the upregulation of Gasdermin‐D (GSDMD) does not inherently confirm the execution of pyroptotic cell death. The investigations of GSDMD immunoreactivity aligned with pTau_217_ and Aβ in the CA2 hippocampal region and suggest that inflammasome signaling is not an age‐related phenomenon. However, it may be due to the progression of AD neuropathological changes, which correlated with gene profiling studies. Regional comparisons across assays were performed in distinct hippocampal subfields (CA1 transcriptomics and CA2 imaging) based on availability of donated brain tissue and methodological constraints. The CA2 region is a highly relevant site of investigation, as its pyramidal cells are heavily populated with NFTs and NPs during the intermediate progression of AD. Our observations that GSDMD immunoreactivity in CA2 aligns with pTau217 and Aβ deposits mirror the transcriptomic trends we identified in CA1, supporting a consistent, region‐wide inflammasome response.

Nearly 25 years have passed since the discovery of the inflammasome. Initially, the inflammasome was characterized as a multiprotein complex responsible for the activation of caspase‐1 and the processing of the pro‐inflammatory cytokines interleukin (IL)‐1β and IL‐18 following infections and autoimmune diseases. Since then, the literature expanded to include sterile inflammatory events like central nervous system (CNS) injury and neurodegenerative diseases such as AD. Currently, the role of the inflammasome is better understood, as a multi‐stage process, and the signaling complexity has been shown to be greater than initially thought, where activation and pore formation are distinct from terminal cell lysis. In addition to its role in the processing of the zymogen caspase‐1 and the processing of IL‐1β, the inflammasome is also involved in the cell death process of pyroptosis through the cleavage of GSDMD. Moreover, what initially were thought of as pyroptosomes, characterized by the oligomerization of the adaptor protein apoptosis‐associated speck‐like protein containing a caspase‐recruitment domain (ASC), are now referred to as ASC specks due to their appearance in fluorescent microscopy images. Interestingly, these ASC specks under electron microscopy images arrange more like filaments.

During inflammasome signaling, caspase‐1 activation can take place independently of pro‐IL‐1β [1] and/or pro‐IL‐18 processing. Similarly, pyroptosis can occur as a result of inflammasome activation, but that does not imply that inflammasome activation will always result in pyroptosis mediated by GSDMD. Moreover, recruitment of the amino terminus of GSDMD to the membrane does not signify that a cell will die by pyroptosis, for ninjurin‐1 (NINJ1), which is involved in the release of lactose dehydrogenase (LDH) and damage‐associated molecular patterns (DAMPs), is needed for plasma membrane rupture and cell lysis, and these effects of NINJ1 can be repaired by endosomal sorting complexes required for transport (ESCRT)‐III [2]. Therefore, when interpreting results regarding inflammasome signaling, it is important to discriminate between the different processes that can happen during inflammasome signaling. The presence of GSDMD cleavage does not imply that a cell will die by pyroptosis if NINJ1 is being inhibited by ESCRT‐III, in which case LDH release would be prevented since inflammasome activation, pore formation, and cell lysis are separate processes. Accordingly, our findings may be interpreted as an inflammasome‐related molecular signature rather than evidence of pyroptotic cell death. Furthermore, caspase‐1 cleavage upon inflammasome assembly does not imply that either or both IL‐1 cytokines will be processed, and caspase‐1 may have its own pathological role in diseases like AD, for it has been shown to associate with tau aggregates [1]. Similarly, the presence of ASC specks in the absence of a full inflammasome complex also suggests signaling due to the prion‐like properties [3] that ASC aggregates have which also aggregates with amyloid‐β (Aβ) [4].

Another layer of complexity arises when two sensors such as NLRP3 and NLRC4 are present in the same cell and may even have redundant roles [5], or when ASC is either present or absent within a complex, for ASC‐containing inflammasomes trigger a more heightened inflammasome response. NLRP3 signal predominates in microglia, NLRP1 in neuronal compartments, and AIM2 in plaque‐associated regions enriched with ASC. Thus, future studies are needed that use super‐resolution confocal microscopy to further elucidate the role that these sensors and the adaptor protein ASC have in inflammasome signaling. Sensor redundancy among NLRP3 and NLRC4 [6], as well as differential ASC dependence, further highlights the complexity of inflammasome signaling.

Inflammasome proteins have been shown to be altered between males and females in rodents [7], and the greater inflammaging present in females is consistent with the exacerbated effects of AD in females when compared to males. Thus, it would be important to carry out histopathological studies powered for the study of sexual dimorphism to better understand how these differences occur in males vs. females in the human brain.

Finally, ASC has been shown to be elevated in the serum of patients with mild cognitive impairment when compared to healthy controls and AD patients [6]. In addition, ASC has been shown to be elevated in patients that present normal cognition a year before cognitive changes arise [8], and ASC immunoreactivity is increased in the CA1 region of the hippocampus [1, 9], an area affected in the early stages of the disease. However, future studies are needed to further validate the role of ASC and other inflammasome proteins in the early, intermediate, and late stages of AD.

Taken together, there are region‐specific areas that present increased inflammasome signaling at the intermediate AD neuropathological stage compared to controls. Furthermore, within those regions affected by advanced AD neuropathology (i.e., Braak stages V–VI) the hallmark signs are designated by the intracellular pTau tangles and extracellular Aβ protein deposits. In the de Rivero Vaccari et al. [1] study, ASC is present in pTau217 positive neurons which supports the potential role of that the inflammasomes has as a therapeutic target to delay dementia related diseases.

## AUTHOR CONTRIBUTIONS

Juan Pablo de Rivero Vaccari, Andrew P. Sawaya contributed to pathway details. David A. Davis and Susanna P. Garamszegi contributed to the interpretation. Xiaoyan Sun, Sakir Humayun Gultekin and Ayled Barreda assisted in the clinical evaluation of the AD pathology. W. Dalton Dietrich, Helen M. Bramlett and Robert W. Keane evaluated inflammasome interpretation. Robert W. Keane assembled the letter. All authors were involved in the preparation and final editing of the manuscript.

## CONFLICT OF INTEREST STATEMENT

Juan Pablo de Rivero Vaccari Helen M. Bramlett, Robert W. Keane, and W. Dalton Dietrich are co‐founders and managing members of InflamaCORE, LLC and have licensed patents on inflammasome proteins as biomarkers of injury and disease as well as on targeting inflammasome proteins for therapeutic purposes. Juan Pablo de Rivero Vaccari Helen M. Bramlett, Robert W. Keane, and W. Dalton Dietrich are scientific Advisory Board Members of ZyVersa Therapeutics. All other authors declare no conflict of interest.

## Data Availability

The data that support the findings of this study are available on request from the corresponding author. The data are not publicly available due to privacy or ethical restrictions.
